# Transcriptional Regulation by Gold(I) Thiolates Involving Interaction With Conserved Cysteine Residues in the Photo-Oncogene Products Jun and Fos

**DOI:** 10.1155/MBD.1994.528

**Published:** 1994

**Authors:** Malcolm L. Handel, Colin K. W. Watts, Richard O. Day, Robert L. Sutherland

**Affiliations:** 1 Cancer Biology Division Garvan Institute of Medical Research and Department of Clinical Pharmacology St Vincent's Hospital Sydney Australia; 2 Department of Cancer Biology Harvard School of Public Health 665 Huntington Ave Boston Ma 02115 USA


### TRANSCRIPTIONAL REGULATION BY GOLD(I) THIOLATES INVOLVING INTERACTION WITH CONSERVED CYSTEINE RESIDUES IN THE PHOTO-ONCOGENE PRODUCTS JUN AND FOS

Malcolm L. Handel#, Colin K. W. Watts, Richard O. Day and Robert L. Sutherland

Cancer Biology Division, Garvan Institute of Medical Research and
Department of Clinical Pharmacology, St Vincent's Hospital, Sydney, Australia
### Present address: Department of Cancer Biology, Harvard School of Public Health,
665 Huntington Ave, Boston, Ma 02115, USA

Jun and Fos are nuclear proteins that form Jun-Jun and Jun-Fos dimers collectively known as activator protein-1 (AP-1). These dimers function as transcription factors by binding to a well defined DNA sequence, the AP-1 site, in the promoters of many pro-inflammatory genes. The DNA binding domains of Jun and Fos are basic regions, rich in lysine (K) and arginine (R), as is usually the case for DNA binding proteins. A remarkable feature of Jun, Fos and a handful of other transcription factors, is the presence of a cysteine (C) residue in the basic domain, forming a KCR sequence. Such an electrostatic environment greatly enhances the ease with which the cysteine residue undergoes oxidation or forms complexes with gold(I). Since it has previously been shown that oxidation of the cysteine prevents DNA binding it was reasonable to suspect that gold(I) salts, by forming a complex with the thiol group of the cysteine residue, might also inhibit AP-1 DNA binding. The consequences of such an action of gold(I) would provide an explanation for a great many of its biological properties. Moreover, the KCR sequence is likely to be a more sensitive target for gold than the majority of potential cysteine targets that do not have this unusual electrostatic environment, thus allowing for some specificity of drug action.

Crude nuclear extracts were incubated with gold(I) thiomalate, gold(I) thioglucose or thiomalic acid, and binding of Jun and Fos to a radiolabelled AP-1 site was determined by electrophoretic mobility shift analysis (EMSA). The gold(I) thiolates, but not thiomalic acid, inhibited AP-1 DNA binding with an IC50 of 3 μM. Inhibition was virtually complete with 10 μM gold(I). This effective concentration is an order of magnitude lower than the inhibitory concentration of gold(I) for the most sensitive enzymes previously reported. Recombinant, truncated Jun and Fos proteins with cysteine to serine mutations in their DNA binding domains were added to nuclear extracts, analyzed in the same way, and found to be resistant to the inhibitory effects of gold(I) thiolates. The AP-1 DNA binding of truncated, recombinant Jun and Fos with the wild type sequence had the same sensitivity to gold(I) as the naturally occurring Jun and Fos in the nuclear extracts. These experiments clearly show that gold(I) thiolates inhibit AP-1 DNA binding *in vitro* through a mechanism involving cysteine residues in the DNA binding domains of Jun and Fos.

A corresponding inhibition of AP-1 mediated transcription in living cells was sought by transfecting cultured MCF-7 cells with an AP-1 responsive reporter gene, pCol-TREx5/tk-CAT. Phorbol ester inducible expression of chloramphenicol acetyl transferase (CAT) was significantly inhibited by 10 μM gold(I) thiomalate in both stable and transient transfection systems. In contrast, a similar reporter gene with AP-2 sites in place of AP-1 sites was not inhibited by 10 μM gold(I) thiomalate when transiently transfected into MCF-7 cells.

These results describe a mechanism that may explain many of the biological actions of gold(I) thiolates. Several other transcription factors, including NF-κB, have cysteine residues in their DNA binding domains and these are also candidate targets for gold(I) action.

